# Efficient Production of Dried Whole Peanut Fruits Based on Infrared Assisted Spouted Bed Drying

**DOI:** 10.3390/foods10102383

**Published:** 2021-10-08

**Authors:** Kaiyang Zhu, Linlin Li, Guangyue Ren, Xu Duan, Weiwei Cao, Caixia Qiu

**Affiliations:** College of Food and Bioengineering, Henan University of Science and Technology, Luoyang 471000, China; zhukaiyangyy@163.com (K.Z.); linlinli2020@126.com (L.L.); duanxu_dx@163.com (X.D.); caoweiwei@haust.edu.cn (W.C.); qiucaixiayy@163.com (C.Q.)

**Keywords:** peanut, infrared assisted spouted bed drying, product quality, energy consumption

## Abstract

The present study is designed to evaluate the effect of infrared assisted spouted bed drying (IR-SBD) on the product quality and energy consumption of whole peanut fruits (including peanut kernels and shells). The dehydration of whole peanuts by means of hot-air drying (HD) and infrared drying (ID) were used as the control groups, and the drying characteristics, energy consumption, microstructure, porosity, hardness and fatty acid content were compared. The results showed that, compared to HD and ID, IR-SBD could reduce the drying time by 40% and 33%, respectively, and reduced energy consumption by 66% and 32%, respectively. During the drying process, the structures of both the peanut shells and peanut kernels underwent significant deformation; specifically, the porosity gradually increased gradually. The maximum porosity value was obtained by the samples dried by means of IR-SBD. Under the three drying conditions, the hardness of the peanut shells first decreased and then increased, while the hardness of the peanut kernels showed a trend of first increasing, then decreasing and finally increasing. Compared to the fresh whole peanuts, the IR-SBD dried samples exhibited a 4.07% decrease in fatty acid. This study shows that IR-SBD is a suitable application for the dehydration process of whole peanuts for the purposes of achieving high-efficiency and -quality production in the industrial sector.

## 1. Introduction

Peanut (*Arachis hypogaea* L.) is a nutritious grain that is high in protein and fat (containing 47–50% fat, 25–30% protein) and is considered an important source of nutrients for humans [1]. Peanut is an important annual oilseed plant belonging to the Leguminosae family, which is grown in both tropical and sub-tropical countries. In addition to using peanuts make oil, as a cheap protein source, peanuts have become a snack due to their unique taste and numerous nutrients. In China, peanut production has been as high as 1799 tons [2]. However, due to the seasonality and high water content of fresh peanut, dehydration operations have become an important unit in the deep processing of peanut in industry.

Sun drying is the most traditional and commonly used dehydration method for peanuts. However, this drying method is limited by weather factors, and the product quality is low, which is inconsistent with the current consumer demand for high-quality dehydrated products [3]. Hot-air drying and heat pump drying are also common dehydration methods in industrial dried peanut production [4,5]. However, due to the relatively low drying efficiency, these methods consume a lot of time and energy resources [6]. To solve the above problems, more and more scholars have proposed the adoption of a combined drying method. Infrared-assisted spouted bed drying (IR-SBD) is a combined drying technology based on far-infrared drying and spouted bed drying. Far-infrared radiation has the advantages of having a good thermal effect and being energy efficient [7,8,9]. Spouted bed systems can provide pneumatic agitation in the food particle drying process. This agitation not only facilitates heat and mass transfer through the rebuilding of a boundary layer at the surface of the particles, but also enhances the energy efficiency of the whole system by shortening the drying time. Duan et al. [10]. used an infrared-assisted spouted bed to perform drying experiments on rose petals; the drying characteristics and drying time under different air outlet temperatures and the wind velocity were evaluated, and the method’s lower energy consumption was attributed to the reduction in the drying time and the decline in the working time of the spray spouted system and the heating system. Li et al. [11]. reported that the higher drying temperature and airflow speed of infrared-assisted spouted bed drying was beneficial to shortening the drying time and that the drying conditions had great effects on the polysaccharides, yield, color, sensory quality, and storage stability of dried yams. The optimal drying conditions for probiotic-enriched Chinese yams were a drying temperature of 40 °C and an airflow speed of 22 m/s. Manshadi et al. [12]. studied the effect of infrared-assisted spouted bed drying on flaxseed, specifically on the quality characteristics of its oil when extracted by different methods. The results showed that increasing the air temperature in the presence of IR increased the drying rate. The peroxide values (PVs) of the IR-SBD samples were higher than those of spouted bed drying (SBD) at the same temperature. In addition, IR treatment did not notably change the fatty acid composition in flaxseed oil.

In addition, there are many peanut drying methods, such as microwave, vacuum, and vacuum freeze dry methods [13,14,15]. Although the above methods have been applied to peanut drying, there are few data about the effects of these drying methods on the quality of whole peanut fruits. Both ID and IR-SBD have been investigated as a potential method to obtain a high-quality dried product, but there are no reports regarding a comprehensive comparison of the effects of the two drying methods on the quality of whole peanut fruits. Especially for IR-SBD, the effect of the drying process on peanut quality is uncertain, and if the infrared radiation action during the drying process is helpful to improve the peanut quality should be deeply investigated.

The purpose of our work is to evaluate the applicability of IR-SBD in whole peanut dehydration, and to investigate the drying efficiency and quality characteristics of whole peanut IR-SBD by comparing this method with HD and ID; finally, this research aims to provide valuable reference for subsequent industrial production.

## 2. Materials and Methods

### 2.1. Materials

Fresh peanuts were obtained from the Zhengyang County (Henan Province, China). The initial moisture content of the fresh whole peanuts was 1.163 g/g (d.b) which was determined by oven drying at 105 °C for 24 h (AOAC 2000) [16].

### 2.2. Drying Methods

HD, ID, and IR-SBD were used to dry fresh whole peanuts, before the start of the experiment, the drying performance of the IR-SBD method was tested at 50 °C, 55 °C, 60 °C, 65 °C, 70 °C, 75 °C and 80 °C. The results are shown in Table 1.

It can be seen from Table 1 that the drying time and energy consumption were different at different temperatures. When the temperature was low, the drying time was long, and the energy consumption was large. However, when the temperature was high, the decrease in energy consumption was not obvious; even when the temperature reached 80 °C, the energy consumption increased again. A possible reason for this is that the power of the infrared radiator became larger, which caused the energy consumption to increase. In addition, according to the research of ZENG et al. [17], the whole peanut fruits could have accelerated the drying process and may have had a high quality at 70 °C. As such, the drying temperature was set as 70 ± 0.5 °C and the three dryers were warmed up for 15 min to reach the set temperature before the drying experiments were commenced in order to achieve the equilibrium temperature; the materials were spread uniformly on the bed (mesh) of a tray dryer. During drying process, the temperature sensor (Pt100, Shenzhen Platinum Electric Technology Co. Ltd., Shenzhen, China) readings were observed to ensure the drying temperature. The sample weight for each drying experimental group was 500 g.

#### 2.2.1. HD Process

The general HD dryer (SHT, Sanxiong Machinery Manufacture Co. Ltd., Shangyu, China), which includes heating and air supply equipment, was used in for the current work, and the power of the equipment was 1.5 kW. The hot-air flow flowed perpendicularly through the bed at a 1 m/s velocity. During the drying process, the weights of the samples were recorded every 30 min from moisture content of 1.16 g/g (d.b) to 0.65 g/g (d.b) and then every 60 min in the later stage until reaching a final moisture content of 0.1 g/g (d.b) [18]. All of the experiments were performed in triplicate.

#### 2.2.2. ID Process

**Figure 1:**
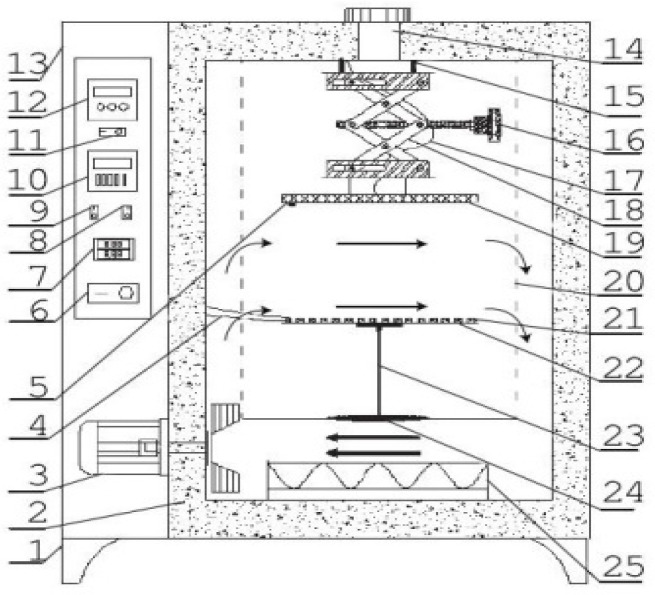
Schematic diagram of infrared radiation drying equipment. (1) Drying chamber stand, (2) thermal insulating layer, (3) fan, (4) thermocouple, (5) thermal sensor, (6) switch, (7) temperature display panel, (8) auxiliary heating switch, (9) fan controller, (10) temperature control panel, (11) infrared radiation heater switch, (12) heater temperature controller, (13) cabinet, (14) exhaust port, (15) tighting screw, (16) knob, (17) heater’s cable, (18) blacket, (19) infrared radiation board, (20) drying chamber, (21) material, (22) material tray, (23) support rod, (24) supporter, (25) electric heater.

The ID equipment used in this research was designed and assembled by the authors [19]. The schematic of the ID equipment is shown in Figure 1. The ID equipment mainly included a drying chamber, an FIR heating system, and a control system. The FIR system mainly consisted of an expansion bracket, an FIR board, and a controller. Two square aluminum oxide plates with side length of 20 cm were set at both ends of the expansion bracket separately. The upper aluminum oxide plate was fixed on the top surface in the drying chamber. A square FIR ceramic board with the size of 18 cm × 18 cm was installed on the surface of the lower plate of the expansion bracket with tightening screws. The FIR board could move up and down by adjusting the lifting knob of the expansion bracket to alter the distance between the FIR board and the materials. The power of the FIR board could be adjusted by a control system and the power value could be read by an electricity meter. A Pt100 thermal sensor was fixed on the surface of the FIR board to measure the surface temperature. Then, approximately 500 g of peanut was uniformly spread onto the tray, and then put into the drying chamber. The drying temperature, radiation distance, and air velocity were set as 70 ± 0.5 °C, 100 mm and 1 m/s, respectively. All of the experiments were performed in triplicate.

#### 2.2.3. IR-SBD Process

**Figure 2:**
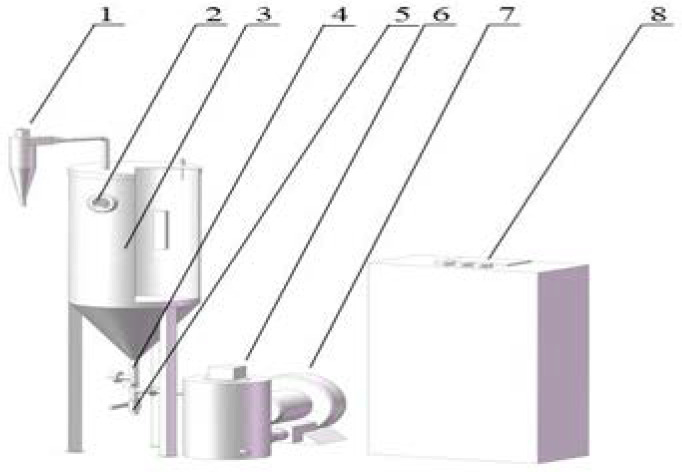
Schematic diagram of IR-SBD device. (1) Cyclone separator, (2) observation window, (3) dry container, (4) temperature sensor, (5) discharge hole, (6) preheating tank, (7) axial flow fan, (8) control system.

In order to protect the quality of the peanuts after harvest and to increase the drying efficiency, a dryer based on spouted bed and variable temperature control technology was built. The schematic of the IR-SBD equipment is shown in Figure 2. The infrared-assisted spouted bed dryer developed by the authors consisted of a frequency conversion controlling centrifugal blower (1 kW with an airflow rate of 220 m^3^/h), drying chamber and four ceramic infrared heaters (3 kW, FSR250W, Elstein-Werk M. Steinmetz GmbH & Co. KG, Germany Hamburg), which were installed at a uniform angle at the middle inner wall of the cylinder. The samples in the fountain region could then be heated by infrared radiation. The drying chamber consisted of a cylinder (diameter and height of 550 mm and 1500 mm) at the top and a conic (diameter and height of 550 mm and 450 mm) at the bottom. The outlet temperature was detected by a Pt100 temperature sensor. The inlet airflow speed was detected by a wind velocity sensor (KA25, Shenyang KANO Scientific Instrument Co., LTD., Shenyang, China).The drying temperature, and inlet wind speed were set as 70 ± 0.5°C and 16 m/s, respectively. During the drying process, aluminum beads were used as inert materials to increase heat transfer in the spouted bed dryer (SBD).The beads had a mean diameter of 15 mm and a round shape, and they weighed 1000 g [12]. The sample weights were recorded every 30 min during the early stage and then every 60 min during in the later stage until reaching the final moisture content was reached. All of the experiments were performed in triplicate.

### 2.3. Drying Characteristics Analysis

The peanut moisture content was calculated according to Equation (1) [20]:

(1) $$X=\frac{m_{t}-m}{m}$$

where, *X* is the moisture content (g/g, d.b), *m_t_* is the weight of the sample at time *t* (g), and *m* is the final weight of the samples (g). Experiments were performed in triplicate.

The peanut drying rate under different drying conditions was calculated according to Equation (2) [21,22]:

(2) $$U=\frac{M_{t_{1}}-M_{t_{2}}}{t_{2}-t_{1}}$$

where, *U* is the drying rate of the material between time *t*_1_ and time *t*_2_ during the drying process, g/(g h); $M_{t_{1}}$ and $M_{t_{2}}$ are the peanut moisture content at time *t*_1_ and time *t*_2_, respectively, g/g (d.b).

### 2.4. Microstructure by Scanning Electron Microscopy

The microstructure of the peanut kernels and shells under different drying conditions was observed using scanning election microscopy (TM3030, Hitachi Co., Tokyo, Japan). Small pieces with dimensions of 5 × 5 × 1 mm were cut from the dried peanut kernels and shells using a sharp razor blade, fixed on aluminum stubs using conductive adhesive tape, and immediately sputtered with gold for 10 nm. The SEM observations were performed at a magnification of 200 times.

### 2.5. Texture Analysis

The hardness changes of the peanut kernels and peanut shells during drying were measured by using a food physical property analyzer (TA.XT Express, Stable Micro Systems., London, UK). Using a cylinder probe (diameter: 2 mm), a uniaxial puncture test, was conducted on single peanut halves, which were individually mounted over a platform. During the test, the sample was placed horizontally under the probe, and three measurements were taken from left to right along the fullest part of the sample, with a distance of 0.5 mm between the two measuring points. Tests lasted until the examined sample was destroyed and until the maximum compressive force (Fcmax) value was determined. The textural parameters of the single peanut halves were expressed as hardness (highest peak compression force (N)). The probe type was P/2 mm, which was a cylindrical stainless steel probe with the diameter of 2 mm. The pre-test rate, test rate, post-test rate, compression degree, and trigger stress were set as 0.8 mm/s, 0.5 mm/s, 0.8 mm/s, 40% and 10 g, respectively. Each test point was repeated three times.

### 2.6. Porosity Measurement

Porosity is the ratio of the pore volume to the total volume of bulk materials in natural state. Peanut porosity was determined from the true and bulk density values of the peanuts. The true density of a peanut and its kernel is defined as the ratio of the mass of a peanut sample and its kernel to the solid volume occupied by the sample. The peanut and kernel volumes and true densities were determined using the liquid displacement method. Toluene (C7H8) was used rather than water because it is absorbed by peanuts and to a lesser extent by their kernels. The surface tension of toluene is low, so it even fills shallow dips in a peanut and its kernel and their dissolution power is low [23]. The bulk density of the peanuts was determined by the ratio of the weight of the peanuts contained in a cylindrical container to the volume of the container.

### 2.7. Fatty Acid Determination

Chromatographic analysis was conducted on a Trace GC Ultra/TSQ Quantum XLS system (TSQ 9000, Thermo Fisher Scientific., Waltham, MA, USA), a gas chromatograph coupled with a mass spectrometer (MS). Oil samples obtained by the Soxtec extraction method were used for fatty acid analysis. About 10–12 mg of peanut oil was reacted with 0.2 mL of sodium methoxide to convert the oil into the corresponding fatty acid methyl esters. The mixture of oil and sodium methoxide was heated to 50 °C and was mixed by sonication for 5 min to complete this transesterification reaction. The esters were recovered after the addition of 0.5 mL of cyclohexane. This mixture was sonicated for about 10–15 min and was allowed to separate into two phases. A 10–15 μL sample was taken from the upper organic phase and was diluted with 1 mL of acetone for GC analysis. Analysis of the calibration solutions and the derivatized extract samples was performed in positive electron impact ionization (EI+) mode. The ion source temperature was 280 °C, andthe oven temperature was programmed at 120 °C for 1 min, and increased to 240 °C with 2.5 °C/min and held for 20 min. The mobile phase was helium of high purity, at a constant flow rate of 1 mL/min. A volume of 1 μL of extract was injected at 240 °C. Instrument control and, data acquisition and processing were performed using the Xcalibur Program. 

### 2.8. Energy Consumption

During drying process, we used three different dryers that were equipped with special electric meters (DDS825, Shanghai People Instrument Co., Ltd. Shanghai, China), and recorded the readings of the meters before and after the measurements, and subtracted the two readings to obtain the electrical energy consumed by the entire equipment during the drying process. As such, the energy consumption in the test could be observed by the electric power consumption.

### 2.9. Statistics Analysis

All of the measurements were performed at least in triplicate, and the results were presented in the figures by their averaged values and error bars representing standard errors (computed from standard deviations and 95% confidence intervals). Statistical analysis was conducted using SPSS software (Version 18.0, SPSS, Inc., Chicago, IL, USA). Significant differences among product quality attributes are expressed with small letters a–c in the bar graphs showing the results. This was obtained by one-way analysis of variance (ANOVA), for significance at the *p* < 0.05 level. Origin 8.5 statistical software (Origin Lab, Northampton, MA, USA) was used for data analysis and processing.

## 3. Results and Discussion

### 3.1. Drying Curves

**Figure 3:**
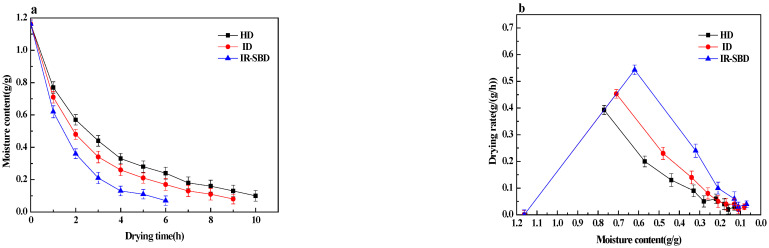
Drying curves (**a**) and drying rate curves (**b**) of whole peanuts under three different drying methods.

The effect of different drying methods on the drying characteristics of fresh whole peanuts is shown in Figure 3a. The drying time required to reach the intended peanut moisture content (0.1 g/g (d.b)) for HD, ID, and IR-SBD was 10, 9 and 6 h, respectively. The effect of different drying methods on the time required for drying whole peanut fruits to achieve a moisture content of 0.1% on a dry basis was significant at the 5% level (Table 2). Compared with HD and ID, the drying time of IR-SBD is reduced by 40% and 33%, respectively. IR-SBD provides pneumatic agitation in the peanut particles drying process and this agitation not only facilitates heat and mass transfer through rebuilding a boundary layer at the surface of particles, but also destroys the peanut shell structures [24], which facilitates the moisture transfer from inside to the outside of the peanut, and results in a higher drying rate.

During the IR-SBD process, fresh whole peanuts are heated more uniformly. This result is consistent with the research results of Serowik et al. [25]. Both results indicate that adding better heating source to the spouted bed could produce a more uniform drying effect. The spouted bed system provides pneumatic stirring for the material particles, which not only promotes mass and heats transfer by reconstructing the boundary layer on the particle surface, but also improves the energy utilization of the entire system. 

The drying rate of fresh whole peanuts under different drying methods is shown in Figure 3b. ANOVA results indicate that different drying conditions have a significant effect on the drying rate of peanuts (*p* < 0.05). It can be concluded that IR-SBD is the most efficient method. During the drying process, there is a significant deceleration stage, indicating that moisture diffusion is controlled by internal diffusion, which determines the rate of the mass transfer process. At the beginning of the drying process, the moisture of fresh whole peanuts is rapidly removed, then a decreasing trend in the drying rate is obvious. Moreover, the drying method is the main factor affecting the drying rate. The drying rate gradually became slower as the moisture content changed from moisture content of 0.3 g/g (d.b) to 0.1 g/g (d.b) for HD, ID and IR-SBD. The possible reason for this is that as the water content of peanuts decreases, the water migration power caused by the difference between internal and external water potential gradually weakened, thus the drying rate decreased. On the other hand, the fact that peanut shells become hard during drying is also a negative aspect of whole peanut dehydration [26,27].

### 3.2. Microstructure Change

**Figure 4:**
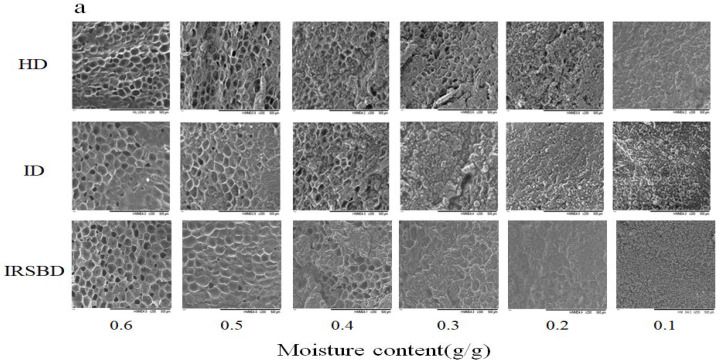

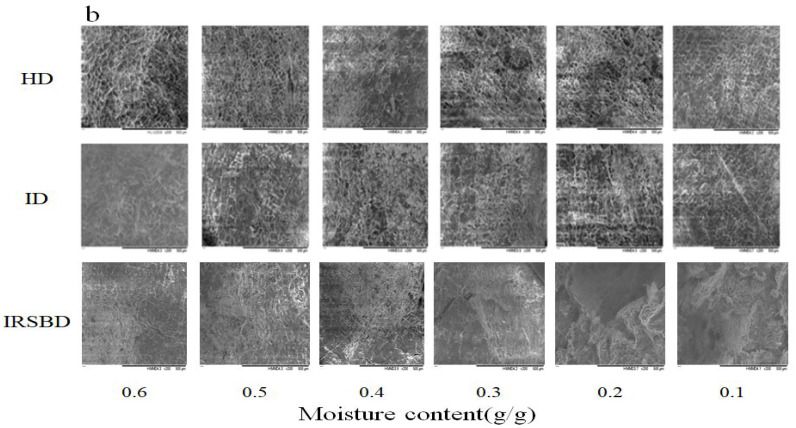
Microstructure observation of peanut kernels (**a**) and peanut shells (**b**) under different drying methods (×200).

To study the influence of the drying method on the peanut microstructure during the drying, the SEM images of the peanut samples during the drying process are captured and are shown in Figure 4. It can be observed that at the early drying stage, the porous structure of the peanut kernels is complete and has a large aperture, as well as a clear and regular arranged cell boundary. During the drying process, the peanut cell aperture gradually decreases, and the peanut structure is closer together. When the moisture content was 0.4 g/g (d.b.), the peanut kernel network structure began to deform, and the surface appeared to have an uneven granular structure. In the late drying stage, the reticular structure of peanut kernels underwent a large amount of deformation, and the granular structure became more prominent. Combined with the analysis of the drying curve in Figure 3a, it can be seen that the changes to the peanut cell structure are closely related to the moisture content, which affects the peanut drying process in real time. Due to the continuous shrinkage of the peanut tissue structure during the drying process, the reticular cell structure gradually became deformed, increasing the water diffusion resistance, which is not conducive to water loss. When the moisture content reached 0.3 g/g (d.b), the network structure of the peanut kernels under going ID were completely deformed, while the network structure of the peanut kernels under going IR-SBD still existed, indicating that IR-SBD can overcome the shortcomings of ID improving drying uniformity.

**Figure 5:**
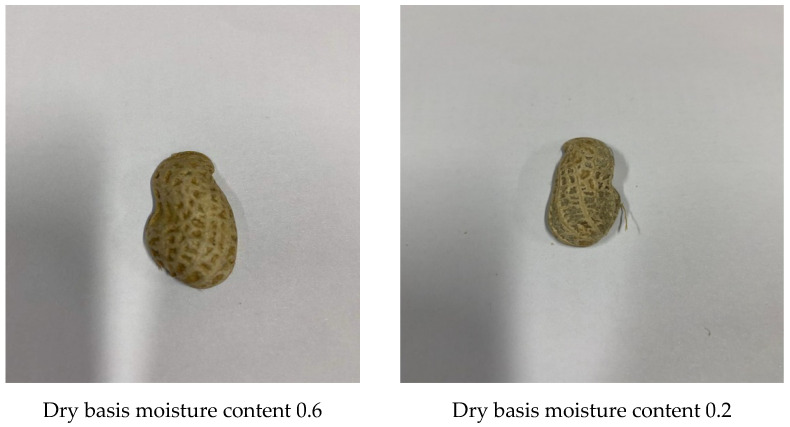
Peanut shells with different dry basis moisture content after IR-SBD treatment.

Figure 4b shows that when the moisture content is decreased to 0.6 g/g (d.b), the structure of peanut shell is loose. During the drying process, the peanut shell structure gradually contracts, and the microstructure become denser, which leads to the water diffusion becoming more difficult within the peanut kernel, reducing the drying efficiency. Comparing the microstructure of peanut shells with the same moisture content under different drying methods, it can be seen that IR-SBD makes the peanut shell produce visible pores in the late drying period. From Figure 5, it can be seen that IR-SBD makes the peanut shell produce pores in the late drying period, which not only improves the drying rate of peanuts with shell, but also increases the peanut porosity.

This may be because the peanut shell itself had a certain protective effect on the peanut kernel. The peanut kernel water loss rate is significantly higher than that of the shell during the drying process. The gap between the shell and the kernel can act as an insulating layer to prevent the migration of water. However, the pores produced by the peanut shell under IR-SBD played a bridge role, which destroyed the integrity of the shell and caused mechanical damage to the shell, so the peanut kernel could be considered to be in direct contact with the outside world. Therefore, IR-SBD has a significant effect on the improved drying rate of peanuts with shells.

### 3.3. Hardness

**Figure 6:**
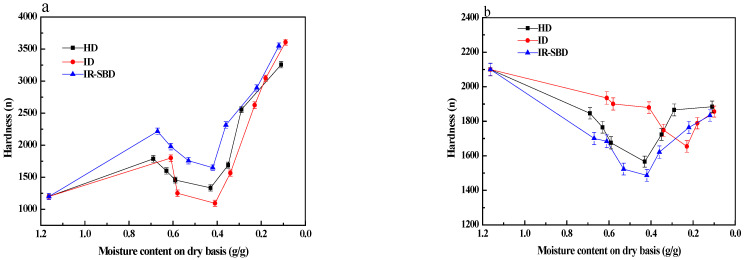
Hardness changes of peanut kernel (**a**) and peanut shell (**b**) during three drying methods.

Figure 6a illustrates that the hardness of peanuts dried by different drying methods and shows a trend of increasing-decreasing-increasing with the decrease in moisture content. As shown in Figure 3b, at the beginning of the drying process, the drying rate of peanuts with shells under IR-SBD is the highest. Therefore, the hardness changes the fastest. As drying continues, the water diffusion in peanut is good at the beginning of drying, so the moisture content decreases, and the hardness increases. In the middle drying stage, the network structure of the peanut kernel is deformed, and the water diffusion channel is blocked. At the same time, the peanut shell also has a certain protective effect, so a high-temperature and humid environment is formed inside the peanut. In this environment, the peanut kernel begins to soften, the hardness decreases, and the toughness increases. In the hardness reduction stage, the hardness of peanut kernels dried under a single infrared heating mode decreased most, which may be due to the fact that infrared heating transmits heat via radiation. When the infrared radiation reaches the material surface, it penetrates the surface and enters the material at a depth of 1–3 mm [28]. The radiation energy is transformed into heat energy, which increases the internal heating temperature of the material, accelerating the moisture migration of the peanut kernel to the outside. However, due to the protective effect of peanut shell, the moisture diffusion on the surface of peanut kernel is prevented and forms a humid environment inside peanut, resulting in the softening of peanut kernel and a sharp decrease in hardness. With the drying progress, the continuous high temperature environment make the humidity around the peanut smaller and smaller, and the hardness of the peanut kernel gradually increases until the end of the drying process.

Figure 6b shows that the hardness of peanut shells first decreases and then in-creases during the drying process. The hardness of fresh peanut shells is the largest because of the high moisture content. The hardness decreases due to the moisture decrease and toughness increase during the drying process. At the early stage of the drying process, the hardness of the peanut shells dried by means of IR-SBD decreased the most, and the hardness of the peanut shells dried by means of ID decreased the least. The ID and IR-SBD methods also produce different hardness behaviors as the drying process proceeds. The possible reason for this is that ID and IR-SBD are both heated by infrared radiation, but the peanuts are undergoing a static drying process under the ID method, and the peanuts are in dynamic drying process under the IR-SBD method. The IR-SBD drying process, destroys the integrity of the shell and causes mechanical damage to the shell, reducing the density of the peanut shell. As the density of the peanut shell reduces, the hardness gradually reduces. ANOVA results (Table 2) indicate that the different drying conditions have no significant effect on the final peanut hardness values (*p* > 0.05). Under the three drying methods of HD, ID, and IR-SBD, the final hardness values of the peanut shells were 1884, 1856, and 1834, respectively. At the end of the drying process, the hardness value of the IR-SBD samples was the smallest. As shown in Figure 3b, a large number of pores are generated in the peanut shells when there is a moisture content of 0.2 g/g d.b, which leads to the hardness of the peanut shells being smaller at this time than it was under the other drying methods.

### 3.4. Porosity

**Figure 7:**
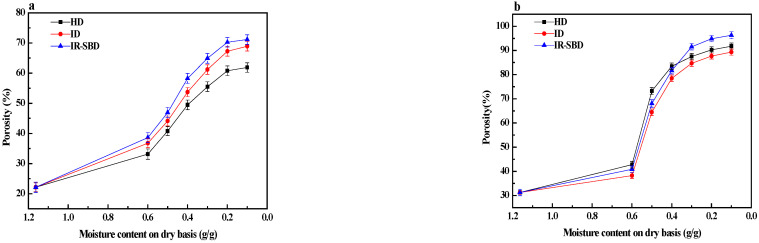
Changes in porosity of peanut kernel (**a**) and peanut shell (**b**) during three drying methods.

Table 2 shows the analysis of variance related to the effect of different experimental methods on porosity. The analysis of variance (Table 2) shows that there was a significant difference between different drying methods at the 5% level. As shown in Figure 7, when the moisture content was 0.1 g/g, the porosities of the peanut kernels were 61.89%, 68.93% and 71.14% when dried by HD, ID and IR-SBD, respectively. When the drying process is over, the porosity of the peanut kernels dried by means of IR-SBD is the largest, while the porosity of the HD sample is the smallest. This might be due to the static drying process of HD, and the way that the material received heat from outside to inside. In the early drying stage, the peanut porosity changed slowly under HD, and this is value smaller than the other drying methods. With the extension of the drying time, the porosity curve of the peanut kernels becomes steep, indicating that the peanut kernels begin to lose a lot of water in the middle drying stage, and the porosity changes rapidly. In the late drying stage, the porosity curves tend to be stable, and the variation amplitude decreases, indicating that the influence of the peanut kernel moisture content on porosity gradually decreases.

### 3.5. Fatty Acids

An examination of the analysis of variance (Table 2) for the factor of fatty acids shows that there is a significant difference between different drying methods at the 5% level (*p* < 0.05).Fatty acid content and composition are important indexes for evaluating the nutritional quality and processing characteristics of peanut kernels, especially unsaturated fatty acids (mainly oleic acid and linoleic acid) in peanuts. The effects of HD, ID and IR-SBD on peanut quality are compared using the fatty acid composition of fresh peanut (FP) as contrast. As shown in Figure 7, after HD, ID and IR-SBD treatment, the decline rates of the total fatty acids are 7.77%, 9.27%, and 4.07%, respectively. The proportion of unsaturated fatty acids in the total fatty acids [29,30] of fresh peanuts is more than 81%. After drying, the unsaturated fatty acid content in peanuts is significantly lower than that in fresh peanuts, the content of saturated fatty acids (SFA) shows no significant change (*p* > 0.05), while the content of unsaturated fatty acids (UFA) decreases. Changes in wind speed, radiation distance, oxygen values and water content during drying can cause different degrees of fatty acid chemical reactions in peanut kernels, especially in terms of unsaturated fatty acids (MUFA) and polyunsaturated fatty acids (PUFA). The stability of MUFA is much lower than that of saturated fatty acids [31], which are very prone to oxidation and loss.

**Figure 8:**
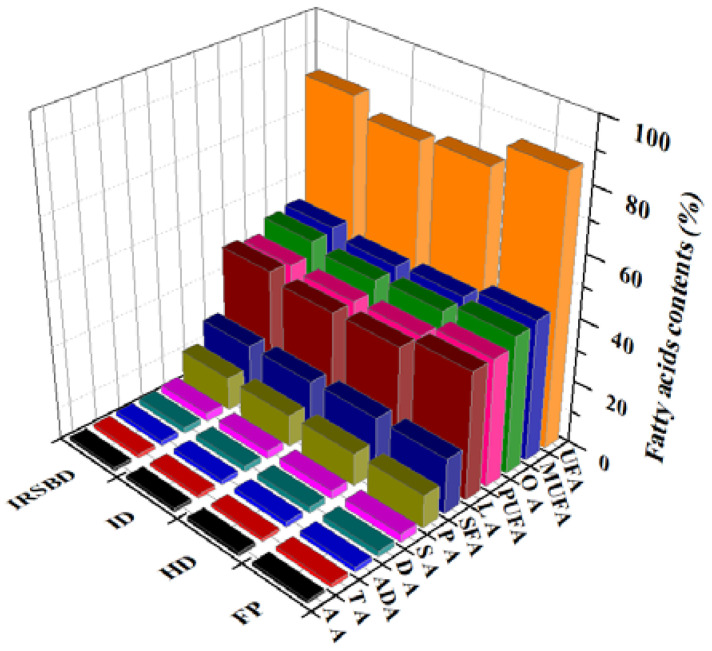
Changes in fatty acids during three drying methods.

As shown in Figure 8, the relative content of main fatty acids in the peanut kernels treated by the three drying methods is in the order of: oleic acid(OA) > linoleic acid(LA) > palmitic acid(PA) > stearic acid(SA) > docosanoic acid(DA) > arachidic acid(ADA) > tetracosanoic acid(TA) > arachenoic acid(AA). Compared to fresh peanut kernels, the decrease in the content of polyunsaturated fatty acids is more severe. This may be because polyunsaturated fatty acids contain more carbon-carbon double bonds, which are more likely to be oxidized and lost. Among the peanuts dried by the three drying methods, the unsaturated fatty acid content of peanuts dried by IR-SBD is relatively higher than that of the other two drying methods, while it is the lowest in the samples dried by means of infrared heating. In the early drying stage, the material is heated rapidly to save the drying time. However, single infrared drying still has the problem of slow dehumidification. The high temperature and high humidity environment cause unsaturated fatty acids to be oxidized more easily [32]. The IR-SBD method combines the advantages of the rapid accumulation of heat inside shelled peanuts and the good uniformity of spouted bed drying, shortening the drying time and reducing the damage to the fatty acids during the drying process. Therefore, the IR-SBD possesses a short drying time and a high unsaturated fatty acid retention.

**Figure 9:**
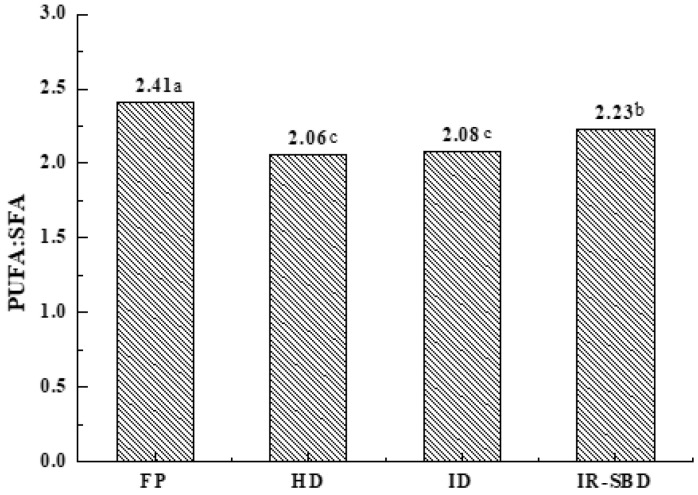
Comparison of the PUFA: SFA ratios to those of untreated fresh whole peanuts. Different letters indicate significant differences (*p* < 0.05).

To further compare the nutritional retention of dried peanut kernels, the ratio of PUFA to SFA (PUFA: SFA) can be estimated [33], which is an important indicator that is used to evaluate the nutritional value of oil. The Chinese Medical Terminology Commission indicates that the PUFA: SFA value should not be less than 1. Figure 9 shows that the PUFA: SFA value of fresh peanuts and dried peanuts are much higher than the recommended value, but the PUFA: SFA value of the dried peanuts is significantly lower that of fresh peanuts (*p* < 0.05), which might be due to the decrease of PUFA that occurs during drying, reducing the nutritional value of peanut oil to some extent. The PUFA: SFA value of the peanut kernels dried used in the IR-SBD method is significantly higher than that of samples dried by other drying methods (*p* < 0.05), indicating the relatively high nutritional value of peanut kernel oil derived from peanuts dried using the IR-SBD method.

### 3.6. Energy Consumption

Drying is one of the most energy-intensive industrial operations and about 7–15% of industrial energy is allocated to this process [34]. Therefore, energy consumption should be considered when the effects of different drying technologies are comprehensively evaluated [35].

**Figure 10:**
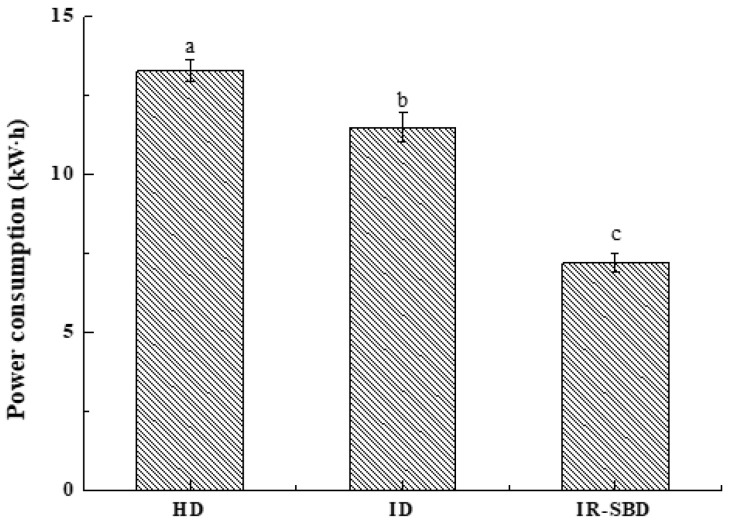
Power consumption of dried peanuts with shells by three drying methods. Different letters indicate significant differences (*p* < 0.05).

Figure 10 shows that the energy consumption of the three drying methods. It can be observed that the electricity consumption of peanut during the HD process is 13.3 kW·h. Under the same conditions, the energy consumption of single ID is 11.5 kW·h. The energy consumption of IR-SBD is 7.2 kW·h, which mainly comes from the axial fan and infrared heating system. Bagheri et al. [36] reported that the rate of energy expenditure for roasting peanut kernels in the infrared roaster. The lowest value of energy consumed while roasting peanut kernels was observed at 200 W for 10 min (0.253 kW·h) and the highest value was at 450 W for 30 min (1.159 kW·h), which means that by choosing the proper roasting conditions, energy consumption can be reduced. Similar findings were reported by Motevali et al. [37] while drying of mushroom slices. The results show that combined drying methods reduced energy consumption up to 45% and 37% to the HD and ID methods studied in our previous research. Therefore, IR-SBD can be considered to be a promising technique for peanut roasting because its of lower energy costs.

## 4. Conclusions

Peanuts usually have high moisture content after harvest and must be dried to avoid mildew. In the current study, through the comparison of different drying methods, the IR-SBD method was confirmed as the best drying method for fresh whole peanuts and shows promising potential for use as an alternative drying technology in the production of dried whole peanut fruits. The trials with whole peanut fruits showed that the drying methods significantly affected the drying characteristics of peanut. The observation of the peanut microstructure shows that during the drying process, the structure of peanut shell and kernel deforms, and the porosity increases, and finally stabilizes. The puncture test showed that the hardness of peanut kernels increases first and, then decreases and finally increases while drying, while the hardness of peanut shells first decreases and then increases. Through physical and chemical analysis, it was found that the fatty acid content in peanut kernels decreases significantly under three drying methods (*p* < 0.05). It can be seen that drying will cause certain damage to the nutrients of the material. The previous studies have proved that drying is a good way to extend the storage time, but the research on what changes the nutrients have caused by drying should be strengthened. Compared to HD and ID, IR-SBD can greatly reduce energy consumption. However, IR-SBD equipment is more complicated and covers a larger area. As such, an economic justification is required for industrial application at a large scale.

An important trend in the modern food industry is the creation of more good foods based on their perceived environmental, health, and ethical benefits. The processing methods used for these products crucial, and finding a new drying method is crucial for the development of the food industry. Through the comparison of different drying methods, the IR-SBD method has been confirmed to be the best drying method for fresh whole peanuts. The findings in the current work provide important information that is necessary for selecting a feasible drying technology for fresh whole peanuts to maximally reduce drying time and, energy consumption, and to preserve product quality attributes. In future study, we think that IR-SBD combined drying technology is worth further research, especially for nut materials. Moreover, whether IR-SBD drying is beneficial for the drying of other vegetables and fruits remains to be seen.

## Figures and Tables

**Table 1:** Comparison of the time and energy consumption of IR-SBD drying whole peanut fruits at different temperatures.

Drying Method	Drying Temperature (°C)	Drying Time (h)	Drying Energy Consumption (kW·h)
IR-SBD	50	10.67 ± 0.32 a	12.84 ± 0.49 a
	55	9.23 ± 0.25 b	11.18 ± 0.39 b
	60	8.34 ± 0.23 c	10.03 ± 0.31 c
	65	7.26 ± 0.23 d	8.71 ± 0.28 d
	70	6.12 ± 0.21 e	7.34 ± 0.25 e
	75	5.50 ± 0.16 f	7.29 ± 0.22 f
	80	5.01 ± 0.12 g	7.87 ± 0.29 g

Values expressed as mean ± standard error (*n* = 3); different letters in each column indicate a significant difference at *p* ≤ 0.05 levels.

**Table 2:** Analysis of variance (ANOVA) for the drying methods on drying time, hardness, porosity, fatty acids, and energy consumption.

		HD	ID	IR-SBD	*p* Value
drying time (h)		10.10 ± 0.32 a	9.11 ± 0.26 b	6.07 ± 0.22 c	<0.05
Hardness(*n*)	peanut kernel	3256.25 ± 206.68 a	3607.22 ± 221.97 b	3548.49 ± 229.65 b	<0.05
	peanut shell	1879.02 ± 69.63 a	1855.15 ± 91.49 a	1831.33 ± 72.04 a	>0.05
Porosity (%)	peanut kernel	61.88 ± 4.85 a	68.81 ± 5.53 b	71.15 ± 4.10 b	<0.05
	peanut shell	91.73 ± 5.64 a	89.33 ± 4.86 b	96.24± 5.92 c	<0.05
Fatty acids (%)		77.85 ± 4.21 a	76.57 ± 4.15 a	82.13 ± 4.35 b	<0.05
Energy consumption (kW·h)		13.31 ± 0.53 a	11.52 ± 0.48 b	7.21 ± 0.23 c	<0.05

Values expressed as mean ± standard error (*n* = 3), different letters in each column indicate a significant difference at *p* ≤ 0.05 levels.
